# Assessment of a deep-learning system for fracture detection in musculoskeletal radiographs

**DOI:** 10.1038/s41746-020-00352-w

**Published:** 2020-10-30

**Authors:** Rebecca M. Jones, Anuj Sharma, Robert Hotchkiss, John W. Sperling, Jackson Hamburger, Christian Ledig, Robert O’Toole, Michael Gardner, Srivas Venkatesh, Matthew M. Roberts, Romain Sauvestre, Max Shatkhin, Anant Gupta, Sumit Chopra, Manickam Kumaravel, Aaron Daluiski, Will Plogger, Jason Nascone, Hollis G. Potter, Robert V. Lindsey

**Affiliations:** 1 Imagen Technologies, Inc., 151 West 26th Street, Suite 1001, New York, NY 10001 USA; 2 Hospital for Special Surgery, 523 East 72nd St, New York, NY 10021 USA; 3 Mayo Clinic, 200 1st St SW, Rochester, MN 55905 USA; 4 University of Maryland Medical System, R Adams Cowley Shock Trauma Center, 22 South Greene Street, Baltimore, MD 21201 USA; 5 Stanford University, 450 Broadway St, Redwood City, CA 94063 USA; 6 The University of Texas Medical School at Houston, 6431 Fannin Street, Houston, TX 77030 USA; 7 University of Maryland Medical System, 22 South Greene Street, Baltimore, MD 21201 USA

**Keywords:** Bone, Software

## Abstract

Missed fractures are the most common diagnostic error in emergency departments and can lead to treatment delays and long-term disability. Here we show through a multi-site study that a deep-learning system can accurately identify fractures throughout the adult musculoskeletal system. This approach may have the potential to reduce future diagnostic errors in radiograph interpretation.

Misdiagnosed fractures are the leading cause of diagnostic errors in Emergency Departments (EDs), occurring in ~1% of all ED patient visits^1–4^. Missed fractures are the most common type of interpretational error made by physicians on musculoskeletal radiographs^4–7^. They can result in treatment delays, may lead to malunion and arthritis with attendant morbidity^2^, and are one of the most common factors leading to malpractice claims against physicians^5,8^.

Reliably identifying fractures on radiographs is difficult because fractures are uniquely heterogeneous: they can occur in any bone and their appearance depends on the regional anatomy and radiographic projection. Clinicians without a specialization in musculoskeletal imaging generally have limited training at identifying fractures across their many distinct and often-subtle presentations. Providing ED clinicians with timely access to the fracture-detection expertise of specialists could help a large number of patients receive more accurate and timely diagnoses and could help address the leading cause of diagnostic errors in EDs.

The primary aim of this study was to build and test a deep-learning system to provide clinicians with the timely fracture-detection expertise of experts in musculoskeletal imaging (see Fig. 1). We developed a deep-learning system for detecting fractures across the musculoskeletal system, trained it on data manually annotated by senior orthopedic surgeons and radiologists, and then evaluated the system’s ability to emulate them. Prior deep-learning systems for fracture detection are limited in scope to single bones, areas within a bone, specific anatomical regions (e.g., refs ^9–11^), or limited clinical settings (e.g., orthopedic settings—hand, wrist, ankle^12^). Deep-learning methods have recently shown great promise at successfully addressing a wide variety of medical visual search tasks^13–16^, but have yet to tackle a common and heterogeneous clinical problem in medical imaging.

The overall AUC of the deep-learning system was 0.974 (95% CI: 0.971–0.977), sensitivity was 95.2% (95% CI: 94.2–96.0%), specificity was 81.3% (95% CI: 80.7–81.9%), positive predictive value (PPV) was 47.4% (95% CI: 46.0–48.9%), and negative predictive value (NPV) of 99.0% (95% CI: 98.8–99.1%). Secondary tests of radiographs with no inter-annotator disagreement yielded an overall AUC of 0.993 (95% CI: 0.991–0.994), sensitivity of 98.2% (95% CI: 97.5–98.7%), specificity of 83.5% (95% CI: 82.9–84.1%), PPV of 46.9% (95% CI: 45.4–48.5%), and NPV of 99.7% (95% CI: 99.6–99.8%).

Over half of the regional anatomies had mean AUCs above 0.98; foot was the lowest-performing with an AUC of 0.888 (95% CI: 0.851–0.922) and knee was the highest performing with an AUC of 0.996 (95% CI: 0.993–0.998) (see Fig. 2). Performance varied by fracture type, with the lowest AUC of 0.948 for fractures without lucent lines (95% CI: 0.931–0.963) and the highest AUC of 0.982 for fractures without callus formations (95% CI: 0.979–0.985) (see Supplementary Table 1).

**Fig. 1:**
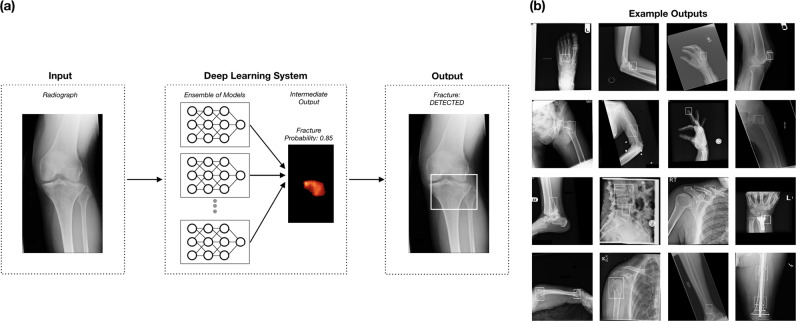
The deep-learning system. **a** The deep-learning system used an ensemble of 10 convolutional neural networks. To produce a prediction, radiographs are processed by each network in the ensemble, averaged, and then post-processed to generate an overall fracture determination and bounding boxes. **b** Example outputs for each of the 16 anatomical regions supported by the deep-learning system.

These results demonstrate that a deep-learning system can accurately emulate the expertise of orthopedic surgeons and radiologists at detecting fractures in adult musculoskeletal radiographs, a challenging heterogeneous clinical problem that is the largest source of diagnostic errors in emergency departments. In each of the 16 anatomical regions tested and across different fracture types (e.g., cases without lucent lines), the deep-learning system has a high AUC, indicating a high level of agreement with the experts’ judgments.

As expected, there was performance variation across anatomical regions, with the lowest AUC in foot. Foot fractures are commonly missed by clinicians^17^ and the foot is one of the most visually complex regions in the musculoskeletal system, with over two dozen bones. Importantly, performance was high across different fracture types ranging from 0.948 for images without lucent lines to 0.982 for images without callus formation. The data suggest that performance of the deep-learning system is robust to clinical variation and to cases that physicians would consider more difficult.

Although deep-learning methods have shown promise at addressing a variety of medical visual search tasks^13–15^, they have yet to tackle a heterogeneous clinical problem in medical imaging such as identifying fractures across 16 anatomical regions. Prior deep-learning studies for fracture detection typically have been limited to a single bone or anatomical region (e.g., wrist or hip^9–11,13^) and the most similar study in scope is a deep-learning model that reports an overall AUC of 0.929 detecting abnormalities in only upper extremity musculoskeletal radiographs^18^. Thus, the present study has much broader clinical breadth than prior deep-learning systems for musculoskeletal radiographs.

This study has limitations. In order to ensure that conclusions could be drawn about the efficacy of the deep-learning system in each anatomical region, we over-represented infrequently acquired regions (e.g., clavicle) relative to more commonly acquired regions (e.g., foot). Thus, in practice, the overall AUC of the system may be different and vary depending on the distribution of anatomical regions. Similarly, the PPV of the system is relatively low (47.4%) due to the low fracture prevalence in the test dataset and the high sensitivity of the system. While our deep-learning system was designed to yield a high sensitivity, further clinical research should assess whether a different balance of sensitivity and PPV (potentially a lower sensitivity and higher PPV) can aid clinicians most effectively. Finally, while the deep-learning system demonstrated high performance on a diverse dataset from two healthcare systems, future research will be necessary to evaluate performance with additional healthcare systems.

As measured by AUC, sensitivity, and specificity, the deep-learning system has a high performance across a wide range of anatomical regions. The results were used to support FDA clearance of a deep-learning system to assist clinicians in detecting fractures for a selected subset of the 16 anatomical regions^19^. Further clinical research is necessary to evaluate the potential of the deep-learning system to reduce diagnostic errors and to improve patient outcomes.

## Methods

### Overall design and testing

To build the deep-learning system, 18 orthopedic surgeons and 11 radiologists manually annotated a model-development dataset of 715,343 de-identified radiographs from 314,866 patients collected from 15 hospitals and outpatient care centers in the United States (see Table 1). Orthopedic surgeons and radiologists were included as annotators as both physicians have expertise in detecting fractures within the musculoskeletal system^20–22^. On each radiograph, a single annotator drew a bounding box that was as small as possible around the site of any clinically relevant fracture (i.e., impactful on subsequent patient care) or noted that the radiograph contained no visible fractures as per specifications in a comprehensive fracture taxonomy. The annotators were provided only with the radiograph and not the original radiologist’s interpretation. The development dataset was randomly split into a training set (80% of the development set), a tuning set (10%), and a validation set (10%).

**Table 1:** Characteristics of the development and test datasets.

	Development dataset	Test dataset
Radiographs		
No. of hospitals^a^	15	15
No. of radiographs	715,343	16,019
No. of radiographic views	16^b^	9^c^
No. of anatomical regions	16	16
Median (range) radiographs per anatomical region	40,658 (6249–106,705)	1000 (774–1079)
No. of radiographs with fracture(s) (%)	82,830 (12%)	2415 (15%)
No. of fracture bounding-box annotations	97,559	2718^d^
No. of bounding-box annotations per fractured radiograph, mean (range)	1.2 (1–13)	1.1 (1–6)
Patients		
No. of patients	314,866	12,746
Median (range) patients per anatomical region	18,952 (3022–71,484)	909 (326–1042)
No. of male (%)	137,929^e^ (44%)	5520 (43%)
Median (range) patient age in years	54 (0–90)^f^	55 (22–90)
Annotators		
No. of orthopedic surgeons (median years experience post-residency)	18 (16)	11 (13)
No. of radiologists (median years experience post-residency)	11 (13)	7 (13)

No radiographs used for testing were in the development dataset.

^a^Datasets sampled from the MedStar Health System located in Baltimore, MD, Washington, D.C., Olney, MD, Leonardtown, MD, and Clinton, MD, the CarePoint Health System in Bayonne, NJ, Jersey City, NJ, and Hoboken, NJ as well as the Hospital for Special Surgery (HSS) in New York, NY and Orthopedic Institute for Children in CA.

^b^Number of unique radiographic views estimated through a manual review of 20,000 randomly sampled radiographs across anatomical regions.

^c^Views were collapsed for statistical analyses into frontal view (frontal; frontal dorso-plantar; frontal inlet-outlet), lateral view (axillary; frog-leg lateral; lateral; y), and oblique view (oblique; oblique-mortise).

^d^2718 reflects unique fracture sites after fusing the 3 reference standard annotations per image through a pixel-wise majority vote.

^e^602 patients were missing biological sex information.

^f^Patient age missing for 43% of the development dataset because patient age was removed from radiographs collected at HSS. De-identification procedures capped patient age at 90 years. In the development dataset, 0.1% of radiographs were from patients 0 to 10 years of age, and 2.95% were from patients 10 to 20 years of age. By design, no radiographs in the test dataset were from patients <22 years of age.

After collecting annotations on the development dataset, we created an ensemble of 10 convolutional neural networks that identifies and localizes fractures from their appearance on radiographs (see Fig. 1). All networks used minor variants of the Dilated Residual Network architecture^23^, with variation introduced in the image pre-processing techniques and the output layer structure. Each network was independently optimized on the training set to predict the probability that a radiograph contained a fracture (“radiograph-level probability”), and, for a subset of networks, the probability that each pixel was part of a fracture site (“pixel-wise probability map”). Network parameters were iteratively updated using the gradient-based optimizer Adam^24^ to minimize the binary cross-entropy loss between network predictions and physician annotations. After training, the output of the ensemble could be obtained by passing a radiograph through each network and averaging the resulting outputs. To enable binary radiograph-level predictions, anatomical-region-specific thresholds were optimized on the tuning set such that the ensemble system yielded a 92.5% sensitivity on each anatomical region. In addition, the tuning set was used to determine thresholds that enabled conversion of the ensemble’s pixel-wise probability maps into bounding-box predictions.

**Fig. 2:**
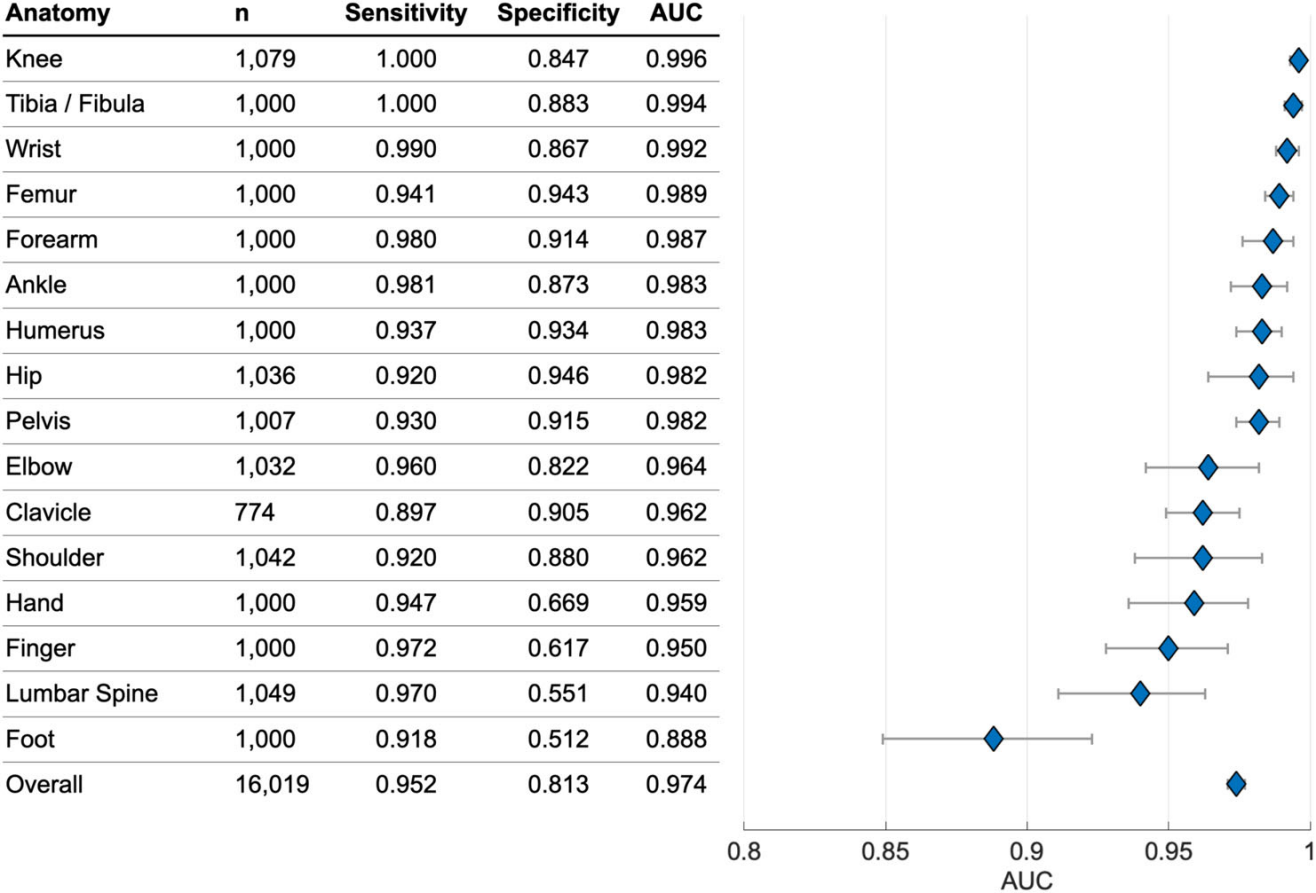
The deep-learning system’s AUCs. Error bars represent 95% confidence intervals calculated using bootstrap sampling (*m* = 1000). *n* indicates the number of radiographs tested.

To test the deep-learning system, we created a test dataset by randomly sampling 16,019 de-identified radiographs from 12,746 adults across 15 hospitals and outpatient care centers. No radiographs from the development dataset were present in the test dataset. Each radiograph in the test set was independently annotated by three orthopedic surgeons or radiologists, without access to the original radiologist’s interpretation. Performance was measured on all 16,019 radiographs, inclusive of the 1265 radiographs where annotators did not agree about the presence or absence of a fracture and a reference standard was constructed using majority opinion (see Table 1). The 14,754 radiographs where annotators agreed about the presence or absence of a fracture were also used to test the performance of the deep-learning system. The main outcome measures were AUC, sensitivity, specificity, positive predictive value (PPV), and negative predictive value (NPV). Confidence intervals for sensitivity and specificity were calculated using Wilson’s method, and all other confidence intervals were calculated using bootstrap sampling (*m* = 1000). Secondary analyses sub-classified positive fracture cases for the presence or absence of a clinical indicator of fracture types, specifically identifying callus formation, displacement, angulation, comminution, and lucent line. Analyses were conducted on cases without each of the indicators to assess performance of the deep-learning system on clinically challenging cases.

### HIPAA compliance

All Protected Health Information used in the training and validation of this deep-learning system was de-identified in compliance with the Healthcare Information Portability and Accountability Act of 1996 (HIPAA)’s Expert Determination method. The de-identification procedures removed DICOM metadata by whitelist-based redaction and transformation of quasi-identifiers. The DICOM pixel data containing PHI was obscured via drawing black boxes over pixel areas containing PHI. These procedures removed all patient information except patient age, institution where the imaging was acquired, and biological sex. The risk that combining these patient variables could disclose the identity of the person was statistically tested with data from the U.S. Census Bureau. The level of re-identification risk was very small and acceptable by HIPAA Expert Determination methods. The study complied with all relevant ethical regulations and a patient waiver of consent was granted by the New England Independent Review Board because the study presented no risk to patients.

### Algorithm design

The deep-learning system’s processing of a given radiograph consists of three stages: a pre-processing stage, the analysis stage, and the post-processing stage.

#### Pre-processing stage

Input radiographs are automatically pre-processed in order to standardize their visual characteristics. A given radiograph is first cropped to remove excess black padding around the edges. Next, an aspect ratio preserving image resizing operation is applied to standardize the resolution to a height and width of 1024px. When necessary in order to preserve the aspect ratio, the resize operation adds padding to the edge of the image. Finally, for use in a subset of the models within the ensemble, the radiograph’s contrast is normalized via Local Contrast Normalization^25^, with the resulting pixel intensities rescaled to lie on the range [−1, 1].

#### Analysis stage

The analysis stage takes the pre-processed radiograph (and its contrast-normalized counterpart) and uses an ensemble of 10 deep convolutional neural networks to create two outputs: one is a score representing the likelihood that any fractures are visible within the radiograph, and the other is a pixel-wise probability map representing an estimate of where any fractures are within the radiograph. All individual models in the ensemble are minor variants of the Dilated Residual Network^23^, which is a type of network that combines the performance benefits of deep residual networks^26^ with dilated convolutions^27^. The models within the ensemble vary in terms of their input (either the pre-processed radiograph, its contrast-normalized version, or both), whether or not the model produces a probability map output in addition to the overall image-level fracture likelihood score, whether or not the model has an attention mechanism, and whether or not spatial pyramid pooling^28^ is used instead of average pooling. Given the fracture likelihood scores from each model, the ensemble’s overall fracture likelihood score is computed by unweighted averaging. Similarly, the ensemble’s probability map output is computed by unweighted averaging over the subset of models that produce probability map outputs.

#### Post-processing stage

The deep-learning system takes the output from the ensemble and applies post-processing operations to create two outputs: a binary determination representing the deep-learning system’s prediction of whether or not any fractures are visible within the radiograph, and a set of bounding boxes associated with any such fracture sites. The binary determination is calculated from the averaged score output from the ensemble using an anatomical-region-specific threshold pre-computed on the tuning dataset. Any score lying on or above the threshold results in a fracture present determination, and any score below the threshold results in a fracture absent determination. The thresholds were optimized to yield 92.5% sensitivity per anatomical region (note that this exact target sensitivity is not necessarily observed on the test dataset). The set of bounding boxes are created from the ensemble’s pixel-wise probability map output using a heuristic that places boxes around the site of high-probability regions within the probability map. The approach also relies on pre-computed, region-specific thresholds that binarize the probability map. The choice to have the deep-learning system output boxes instead of the probability map was based on feedback from physicians suggesting that boxes provide greater clinical utility than probability maps. The choice to compute region-specific binarization thresholds instead of a common threshold was made in order to ensure a minimum sensitivity per anatomical region. Due to the varying incidence rates across anatomical regions, determining a common binarization threshold would result in anatomical regions with more positives dominating the threshold determination.

### Model training

Per standard machine learning practices, the development dataset was randomly subdivided into a “training” (80%), “tuning” (10%), and “validation” set (10%).

Training of each model within the ensemble was achieved by minimizing a joint loss function assessing the model’s ability to correctly predict the image-level classification (fracture present or absent) and the ability to correctly predict the localization of fracture sites. The joint loss function is defined as a weighted sum of two terms. The first term is the average per-pixel binary cross-entropy loss between the predicted probability map and the ground truth map for radiographs with a fracture present. The second term is a binary cross-entropy loss for the image-level classification score. The weights associated with the two terms in the weighted sum are in the ratio (localization:classification) of 3:1 (default) or 10:1 (for models within the ensemble with an attention layer). No weight regularization was used. Data augmentation was used during training; radiographs were randomly rotated, vertically or horizontally flipped, gamma-corrected, contrast-adjusted, vertically or horizontally translated, and zoomed in or out. Due to the low prevalence of fractures in certain anatomical regions, dataset balancing was employed during the first 10 epochs of training to ensure that, on expectation, each possible combination of anatomical region and fracture present/absent label was sampled with equal probability.

Once the parameters of the module were initialized at random (no transfer learning was used), the training algorithm repeatedly iterated through the training set in randomized batches of 48 (default) or 32 (models with attention layer) radiographs. The parameters of the module were updated after processing each batch to minimize the above-mentioned loss function. This minimization was achieved using a variant of the stochastic gradient descent algorithm called Adam^24^. After each epoch, the module was evaluated on the tuning set. Early stopping was used to terminate the training either when the module’s performance on the tuning set did not show any improvement for 10 epochs or when 30 training epochs had been completed. The early stopping performance criteria is the AUC across all radiographs in the tuning set.

After training finished, the radiographs in the validation set were run through the trained ensemble. Internal performance tests were run on the held-out validation set and based on cross-validation on the combined tuning and validation set. For data efficiency, prediction scores on images of the combined tuning and validation sets were used to compute the per anatomical region operating point for the final model. The resulting decision thresholds (one per anatomical region) are then fixed and held constant prior to testing.

### Test dataset

A holdout set of 16,019 unique radiographs was used in this study to test the performance of the deep-learning system, with the main outcome measures reported on the subset of 14,754 radiographs that have an unambiguous reference standard. The 16,019 radiographs were randomly subsampled from a large holdout set composed of radiographs from 15 hospitals within 2 large health systems. The holdout set includes radiographs from the natural distribution in emergency, inpatient, and outpatient settings. From the CarePoint Health system radiographs, holdout radiographs were randomly sampled over a 3.5 year period (January 1, 2013–December 5, 2017). The timeframe of this set overlapped temporally with that of the development dataset, however, it was split prior to development to ensure that the sets of radiographs were disjoint. From the MedStar Health system, the holdout set was composed of 6 months of consecutively sampled radiographs (April 1, 2017–September 31, 2017). The timeframe of this set did not overlap with that of the Medstar Health system data in the training set. Radiographs subsampled for inclusion in the test dataset were manually confirmed to be of a frontal, lateral, or oblique view. The sampling procedure was designed to create a test dataset for each anatomical region containing ~1000 radiographs, enriched as needed to contain at least 100 fractures per region according to a majority vote of 3 annotators. The sampling process was designed to ensure that the set of non-fractured radiographs and the set of fractured radiographs within each anatomical region are each random sample from the larger pool of radiographs.

The hospitals and outpatient care centers in the test dataset are a random subset of those in the development dataset. No radiographs overlapped between the datasets, and 99.98% of test patients (12,744 of 12,746) were not present in the development dataset. The two patients (0.016%) who were present had a radiograph in the development dataset and a different radiograph in the test dataset. The overlap in hospitals and outpatient care centers allows for the possibility that the generalizability of the model’s performance could be affected by the presence of confounding variables specific to the hospitals^29^. This concern is mitigated by training the deep-learning system on bounding-box annotations and training it to produce localized output. Localized training data and model outputs provide interpretability in the model predictions and penalize the model during the training process for being unable to identify the precise location of a fracture in a radiograph predicted to have one. In addition, the models were trained on a diverse dataset of 314,866 patients from 15 healthcare centers, increasing the applicability of the models for new patients.

Further data are provided in Supplementary Tables 1–5 in Supplementary Information.

### Reporting summary

Further information on research design is available in the Nature Research Reporting Summary linked to this article.

## Supplementary information

Supplementary Information

Reporting Summary Checklist

## Data Availability

The output of the model and the ground truth labels used to calculate the results in this study are available upon reasonable request. Access to the X-ray images are not publicly available under Imagen Technologies’ license.

## Code availability

The code used for training the models has a large number of dependencies on internal tooling and its release is therefore not feasible. However, all experiments and implementation details are described thoroughly in the “Methods” so that it can be independently replicated with non-proprietary libraries.
