# Supplementary material for: Assessment of a deep-learning system for fracture detection in musculoskeletal radiographs
Source: NPJ Digit Med. 2020 Oct 30;3:144. doi: 10.1038/s41746-020-00352-w (PMC7599208; doi:10.1038/s41746-020-00352-w)
Supplement: Supplementary file 1 — Supplementary Information [file 41746_2020_352_MOESM1_ESM.pdf]

Supplementary Information

Supplementary Tables

Fracture Types	N obs.	N Fracture Cases	AUC	95% Bootstrap CI
Images without Callus Formation	11608	1535	0.982	(0.979, 0.985)
Images without Displacement	10670	597	0.971	(0.964, 0.977)
Images without Angulation	11511	1438	0.979	(0.975, 0.983)
Images without Comminution	11434	1361	0.978	(0.973, 0.982)
Images without Lucent Line	10292	219	0.948	(0.931, 0.963)

Supplementary Table 1: AUC of the deep-learning system on all 16,019 test radiographs stratified by fracture type.

Anatomy	Sensitivity		Specificity	
	Estimate	95% Wilson's CI	Estimate	95% Wilson's CI
ankle	0.981	(0.946, 0.994)	0.873	(0.848, 0.894)
clavicle	0.897	(0.859, 0.926)	0.905	(0.875, 0.929)
elbow	0.960	(0.902, 0.984)	0.822	(0.796, 0.845)
femur	0.941	(0.891, 0.968)	0.943	(0.926, 0.957)
finger	0.972	(0.936, 0.988)	0.617	(0.584, 0.65)
foot	0.918	(0.852, 0.956)	0.512	(0.48, 0.545)
forearm	0.980	(0.95, 0.992)	0.914	(0.892, 0.931)
hand	0.947	(0.895, 0.974)	0.669	(0.637, 0.7)
hip	0.920	(0.85, 0.959)	0.946	(0.929, 0.958)
humerus	0.937	(0.898, 0.961)	0.934	(0.915, 0.95)
knee	1.000	(0.962, 1)	0.847	(0.823, 0.868)
lumbar spine	0.970	(0.915, 0.99)	0.551	(0.519, 0.582)
pelvis	0.930	(0.862, 0.966)	0.915	(0.895, 0.932)
shoulder	0.920	(0.85, 0.959)	0.880	(0.858, 0.899)
tibia / fibula	1.000	(0.973, 1)	0.883	(0.86, 0.903)
wrist	0.990	(0.963, 0.997)	0.867	(0.842, 0.889)

Supplementary Table 2: Sensitivity and specificity of the deep-learning system on all 16,019 test radiographs.

Anatomy	View	n	AUC	95% Bootstrap CI
ankle	frontal	305	0.988	(0.977, 0.996)
	lateral	367	0.989	(0.972, 0.999)
	oblique-mortise	328	0.975	(0.950, 0.991)
clavicle	frontal	774	0.962	(0.948, 0.974)
elbow	frontal	626	0.971	(0.952, 0.986)
	lateral	406	0.954	(0.906, 0.986)
femur	frontal	543	0.993	(0.987, 0.997)
	lateral	457	0.984	(0.969, 0.994)
finger	frontal	394	0.952	(0.907, 0.985)
	lateral	407	0.941	(0.908, 0.969)
	oblique	199	0.964	(0.920, 0.993)
foot	frontal-dorsoplantar	350	0.896	(0.838, 0.946)
	lateral	353	0.864	(0.782, 0.931)
	oblique	297	0.901	(0.838, 0.956)
forearm	frontal	474	0.984	(0.963, 0.996)
	lateral	526	0.991	(0.982, 0.997)
hand	frontal	307	0.987	(0.967, 0.998)
	lateral	365	0.899	(0.835, 0.959)
	oblique	328	0.973	(0.945, 0.993)
hip	frog-leg	565	0.967	(0.911, 0.998)
	frontal	471	0.987	(0.975, 0.996)
humerus	frontal	509	0.988	(0.978, 0.996)
	lateral	491	0.978	(0.963, 0.989)
knee	frontal	532	0.995	(0.992, 0.998)
	lateral	547	0.997	(0.994, 1.000)
lumbar spine	frontal	360	0.971	(0.938, 0.992)
	lateral	558	0.911	(0.867, 0.951)
	oblique	131	0.991	(0.976, 1.000)
pelvis	frontal-inlet-outlet	1007	0.982	(0.973, 0.989)
shoulder	axillary	65	0.993	(0.970, 1.000)
	frontal	695	0.967	(0.939, 0.989)
	y	282	0.950	(0.895, 0.987)
tibia / fibula	frontal	502	0.994	(0.989, 0.998)
	lateral	498	0.995	(0.990, 0.999)
wrist	frontal	355	0.996	(0.990, 0.999)
	lateral	364	0.993	(0.986, 0.999)
	oblique	281	0.989	(0.977, 0.997)

Supplementary Table 3: AUC of the deep-learning system on all 16,019 test radiographs, stratified by anatomical region and radiographic view.

Demographic / Clinical Characteristic	Level	n (%)
Age Group	22- 44	4951 (30.9%)
	45-64	5944 (37.1%)
	65-74	2391 (14.9%)
	75+	2733 (17.1%)
Sex	F	8991 (56.1%)
	M	7015 (43.8%)
	O	13 (0.1%)
Cast	Absent	15709 (98.1%)
	Present	310 (1.9%)
Fixation Hardware	Absent	14674 (91.6%)
	Present	1345 (8.4%)
Arthroplasty	Absent	15363 (95.9%)
	Present	656 (4.1%)
Confounding Finding[1]	Absent	16,004 (99.9%)
	Present	15 (0.1%)

Supplementary Table 4: Additional summary statistics of all 16,019 test radiographs.

[1] Confounding finding was defined as a medical or radiographic complication that renders a radiograph inconsistent with the typical presentation of pathology and/or impairs or significantly impacts the ability to make a confident diagnosis (e.g., obstructing jewelry, obstructing anatomy, or congenital deformities).

Anatomy	n	Number of Expert Annotators Indicating Fracture				Adjudicated Reference Standard (Majority)
		0	1	2	3	Fracture
Overall	16019	12845 (80.2%)	759 (4.7%)	506 (3.2%)	1909 (11.9%)	2415 (15.1%)
ankle	1000	756 (75.6%)	84 (8.4%)	33 (3.3%)	127 (12.7%)	160 (16.0%)
clavicle	774	384 (49.6%)	69 (8.9%)	39 (5.0%)	282 (36.4%)	321 (41.5%)
elbow	1032	864 (83.7%)	68 (6.6%)	32 (3.1%)	68 (6.6%)	100 (9.7%)
femur	1000	828 (82.8%)	20 (2.0%)	26 (2.6%)	126 (12.6%)	152 (15.2%)
finger	1000	760 (76.0%)	63 (6.3%)	52 (5.2%)	125 (12.5%)	177 (17.7%)
foot	1000	811 (81.1%)	79 (7.9%)	56 (5.6%)	54 (5.4%)	110 (11.0%)
forearm	1000	764 (76.4%)	37 (3.7%)	15 (1.5%)	184 (18.4%)	199 (19.9%)
hand	1000	814 (81.4%)	54 (5.4%)	41 (4.1%)	91 (9.1%)	132 (13.2%)
hip	1036	921 (88.9%)	15 (1.4%)	21 (2.0%)	79 (7.6%)	100 (9.7%)
humerus	1000	724 (72.4%)	39 (3.9%)	22 (2.2%)	215 (21.5%)	237 (23.7%)
knee	1079	964 (89.3%)	17 (1.6%)	24 (2.2%)	74 (6.9%)	98 (9.1%)
lumbar spine	1049	911 (86.8%)	39 (3.7%)	31 (3.0%)	68 (6.5%)	99 (9.4%)
pelvis	1007	863 (85.7%)	44 (4.4%)	29 (2.9%)	71 (7.1%)	100 (9.9%)
shoulder	1042	895 (85.9%)	47 (4.5%)	36 (3.5%)	64 (6.1%)	100 (9.6%)
tibia / fibula	1000	828 (82.8%)	35 (3.5%)	17 (1.7%)	120 (12.0%)	137 (13.7%)
wrist	1000	758 (75.8%)	49 (4.9%)	32 (3.2%)	161 (16.1%)	193 (19.3%)

Supplementary Table 5: Summary statistics of each anatomical region's reference standard on all 16,019 test radiographs.
