# Supplementary material for: Assessment of a deep-learning system for fracture detection in musculoskeletal radiographs
Source: NPJ Digit Med. 2020 Oct 30;3:144. doi: 10.1038/s41746-020-00352-w (PMC7599208; doi:10.1038/s41746-020-00352-w)
Supplement: Supplementary file 2 — Reporting Summary Checklist [file 41746_2020_352_MOESM2_ESM.pdf]

nature research

Corresponding author(s): Robert V. Lindsey

Last updated by author(s): Jul 14, 2020

Reporting Summary

Nature Research wishes to improve the reproducibility of the work that we publish. This form provides structure for consistency and transparency in reporting. For further information on Nature Research policies, see our Editorial Policies and the Editorial Policy Checklist.

Statistics

For all statistical analyses, confirm that the following items are present in the figure legend, table legend, main text, or Methods section.

n/a	Confirmed	
☐	☒	The exact sample size (n) for each experimental group/condition, given as a discrete number and unit of measurement
☐	☒	A statement on whether measurements were taken from distinct samples or whether the same sample was measured repeatedly
☐	☒	The statistical test(s) used AND whether they are one- or two-sided<br>*Only common tests should be described solely by name; describe more complex techniques in the Methods section.*
☒	☐	A description of all covariates tested
☒	☐	A description of any assumptions or corrections, such as tests of normality and adjustment for multiple comparisons
☐	☒	A full description of the statistical parameters including central tendency (e.g. means) or other basic estimates (e.g. regression coefficient) AND variation (e.g. standard deviation) or associated estimates of uncertainty (e.g. confidence intervals)
☒	☐	For null hypothesis testing, the test statistic (e.g. F, t, r) with confidence intervals, effect sizes, degrees of freedom and P value noted<br>*Give P values as exact values whenever suitable.*
☒	☐	For Bayesian analysis, information on the choice of priors and Markov chain Monte Carlo settings
☒	☐	For hierarchical and complex designs, identification of the appropriate level for tests and full reporting of outcomes
☒	☐	Estimates of effect sizes (e.g. Cohen's d, Pearson's r), indicating how they were calculated

Our web collection on statistics for biologists contains articles on many of the points above.

Software and code

Policy information about availability of computer code

Data collection	The code used for training the models has a large number of dependencies on internal tooling and its release is therefore not feasible. However, all experiments and implementation details are described thoroughly in the Methods so that it can be independently replicated with non-proprietary libraries.
Data analysis	The data were analyzed using R version 3.6.1 (2019-07-05).

For manuscripts utilizing custom algorithms or software that are central to the research but not yet described in published literature, software must be made available to editors and reviewers. We strongly encourage code deposition in a community repository (e.g. GitHub). See the Nature Research guidelines for submitting code & software for further information.

Data

Policy information about availability of data

All manuscripts must include a data availability statement. This statement should provide the following information, where applicable:

- Accession codes, unique identifiers, or web links for publicly available datasets
- A list of figures that have associated raw data
- A description of any restrictions on data availability

The output of the model and the ground truth labels used to calculate the results in this study are available upon reasonable request. Access to the x-ray images requires permission from the respective healthcare providers, and so it is not publicly available.

Field-specific reporting

Please select the one below that is the best fit for your research. If you are not sure, read the appropriate sections before making your selection.

☐ Life sciences ☒ Behavioural & social sciences ☐ Ecological, evolutionary & environmental sciences

For a reference copy of the document with all sections, see nature.com/documents/nr-reporting-summary-flat.pdf

Behavioural & social sciences study design

All studies must disclose on these points even when the disclosure is negative.

Study description	The study consists of de-identified patient musculoskeletal radiographs.
Research sample	The musculoskeletal radiographs for the test dataset were from 15 outpatient and healthcare systems in the United States.
Sampling strategy	For the test dataset, the radiographs were randomly subsampled from a large holdout set composed of radiographs from 15 hospitals within 2 large health systems. The holdout set includes radiographs from the natural distribution in emergency, inpatient, and outpatient settings. The sampling procedure was designed to create a test dataset for each anatomical region containing approximately 1,000 radiographs, enriched as needed to contain at least 100 fractures per region according to a majority vote of 3 annotators. The sampling process was designed to ensure that the set of non-fractured radiographs and the set of fractured radiographs within each anatomical region are each random samples from the larger pool of radiographs.
Data collection	Data was collected from 2 large health systems in the United States that consisted of 15 outpatient and healthcare systems.
Timing	CarePoint Health System data sampled over a 3.5 year period (January 1, 2013 - December 5, 2017) and MedStar Health System sampled over a 6 month period (April 1, 2017 - September 31, 2017).
Data exclusions	N/A
Non-participation	N/A
Randomization	N/A

Reporting for specific materials, systems and methods

We require information from authors about some types of materials, experimental systems and methods used in many studies. Here, indicate whether each material, system or method listed is relevant to your study. If you are not sure if a list item applies to your research, read the appropriate section before selecting a response.

Materials & experimental systems

n/a	Involved in the study	
☒	☐	Antibodies
☒	☐	Eukaryotic cell lines
☒	☐	Palaeontology and archaeology
☒	☐	Animals and other organisms
☒	☐	Human research participants
☒	☐	Clinical data
☒	☐	Dual use research of concern

Methods

n/a	Involved in the study	
☒	☐	ChIP-seq
☒	☐	Flow cytometry
☒	☐	MRI-based neuroimaging
